# Using the Griffiths Mental Development Scales to Evaluate a Developmental Profile of Children with Autism Spectrum Disorder and Their Symptomatologic Severity

**DOI:** 10.1007/s10578-022-01390-z

**Published:** 2022-06-28

**Authors:** Maria Chiara Pino, Ilenia Le Donne, Roberto Vagnetti, Sergio Tiberti, Marco Valenti, Monica Mazza

**Affiliations:** 1 Department of Biotechnological and Applied Clinical Sciences (DISCAB), University of L’Aquila, Via Vetoio, Località Coppito, 67100 L’Aquila, Italy; 2 Abruzzo Region Health System, Reference Regional Centre for Autism, L’Aquila, Italy

**Keywords:** Autism spectrum disorder (ASD), Development, Diagnosis, Intervention

## Abstract

Early diagnosis is crucial for Autism spectrum disorder (ASD) and is achieved through a screening of developmental indicators to recognise children who are at risk of autism. One of the most widely used instruments in clinical practice for assessing child development is the Griffiths Mental Development Scale (GMDS). We sought (a) to assess longitudinally whether children diagnosed with ASD, with a mean age of 33.50 months (*SD* 7.69 months), show a developmental delay of abilities measured by the GMDS over time and (b) to analyse which skills of the GMDS could be associate to the symptomatologic severity of ASD. Our results showed lower scores of General Quotient and all sub-quotients of GMDS from first (T0) to second assessment (T1), except for the Performance sub-quotient. Three sub-quotients (Personal-Social, Hearing and Language and Practical Reasoning) also associate symptom severity at the time when the diagnosis of ASD is made.

## Introduction

Autism spectrum disorder (ASD) is a heterogeneous neurodevelopmental disorder characterised by range of atypical social interactions, communication difficulties, repetitive and stereotyped behaviour and restricted interests [1–3]. Its prevalence in the general population is around 1%, with a male–female ratio of 2.5:1 [2–4]. The term spectrum underlines the dimensional nature of the characteristics of the disorder as well as the differences in severity and in the presentation of symptoms of developmental changes [2, 5, 6]. ASD is a life-long condition and a reliable diagnosis is usually not achieved before the age of 3 [1, 7, 8]. Early screening for ASD in primary care is becoming increasingly common and is believed to be a pivotal step towards early treatment. Research into the early recognition and diagnosis of ASD is becoming crucial, so much so that the emergence of ASD symptoms in the first 2 years of life has been well documented [9–12]. This increased focus on early diagnosis and intervention has also led to increased knowledge about the emergence of ASD symptoms and the developmental trajectories of children diagnosed at various ages [13].

Generally, the onset of behavioural and cognitive signs is conceptualised as occurring in one of two ways: as an early impairment and deviation in social and communicative development, or as a period of typical development followed by a marked loss of previous abilities [9, 14]. A third model has been proposed in conceptualising the symptomatic onset of ASD—that is, a developmental stagnation or plateau [15], which is characterised by intact initial skills that do not progress. This pattern of onset differs from regression in that the child does not lose acquired skills but fails to make the expected progress [14]. Several studies have reported that a subset of children initially diagnosed with ASD improve moderately, while other individuals show persistent and severe ASD symptoms; still others show symptoms that worsen during the early period of development [9, 16, 17]. A key to understanding the early emergence of symptoms, however, is to focus on social behaviours that are usually present in early childhood, such as social interest, shared affection, looking at faces and eyes and responding to one’s name [14]. Impairment of social behaviour is the core of this condition [13, 18] and the presence of these markers is also supported by other evidence and appears to be present as early as the first 2 years of life [7, 11, 19–24]. There are also early markers that are not limited to the social domain and may be evident even earlier. In fact, high-risk children who are later diagnosed with ASD show reduced motor control and a delay in fine motor skills at 6 months [25–27]. In addition, evidence suggests that modifying emerging atypical developmental trajectories, through specific interventions, leads to improved prognosis [28, 29].

Assessment of child development through screening of developmental indicators allows the recognition of children who are delayed or at risk of delay and the structuring of early intervention programmes. One of the aims of such assessment is to promote cognitive development to minimise later difficulties and delays. More importantly, the relative strengths and weaknesses of children can be identified at this stage. One of the most widely used instruments in clinical practice for assessing child development is the Griffiths Mental Development Scale (GMDS; [30, 31]). The main advantage of the GMDS is the ability to discriminate different degrees of motor and cognitive development by detecting how much the individual child’s skills deviate from those of the reference sample. This scale can help by indicating at what age a typical child would be able to complete the items, as well as by identifying elements in the domains that have not been achieved [32].

The GMDS has been used to test the concurrent validity of other developmental instruments [33, 34] or as an outcome measure in studies on the predictive value of early cognitive indices [34] and studies on the predictive value of Griffiths’ scales with respect to later development at preschool and school age [35–37]. There is, however, conflicting evidence on the predictive validity of the GMDS [38]. Li et al. [39] recently used the GMDS with an adaptation to the local Chinese culture to delineate a developmental level in children with ASD, with the aim of investigating the existence of cognitive non-homogeneity and analysing the correlation between developmental level and ASD severity (measured by the Childhood Autism Rating Scale; CARS), gender and age at which ASD was first diagnosed. Although their results [39] would represent an attempt to provide a theoretical basis for the intervention strategy, these do not represent a development trend. Further longitudinal studies are needed to investigate the trajectories of development of cognitive domains in ASD to improve the early diagnostic procedure, monitoring, and creation of specific habilitation programs.

In addition, assessing children with ASD over time would allow a developmental trajectory to be outlined to identify whether specific cognitive skills are associated with the symptomatology characteristic of ASD. Indeed, the literature in ASD [9–13] reports an association between level of development and subsequent diagnosis.

Our study, compared to the research by Li et al. [39], represents a deepening in that we used the GMDS scores obtained over time in repeated assessments to monitor the trend of developmental skills. We hypothesised that the main cognitive developmental stages of children at an early age, at risk of or suspected of ASD, affect the severity of symptoms at first diagnosis. For this reason, we implemented a longitudinal observational cohort study, using the GMDS to assess cognitive development presented by children at risk or with a suspected diagnosis of ASD and the Autism Diagnostic Observation Schedule—Version 2 (ADOS-2; [40]) scores for confirmation of the diagnosis. Generally, this research aims to understand the developmental changes in cognitive ability over the course of 1 year, comparing two times: first versus second assessment (T0 vs T1), and the association between facets of cognitive ability and autism symptom severity. Specifically, we focused on two aims: (a) to assess longitudinally whether children diagnosed with ASD, with a mean age of 33.50 months (*SD* 7.69 months), show a developmental delay of skills measured by the GMDS and (b) to explore which skills developmental have an association with ASD symptom severity. Our study could provide support to create early and targeted habilitation programmes for children with ASD.

## Method

### Research Design and Procedure

We implemented a longitudinal observational cohort study. For both assessment times (T0 and T1), the same measures were used, 12 months apart: GMDS and ADOS-2. The investigation was approved by the Ethics Committee of the NHS Local Health Unit and was conducted according to the principles established by the Declaration of Helsinki. All subjects and caregivers were informed about the purpose of the study and the confidentiality of data processing. The subjects’ parents or tutors-by-law provided written informed consent to participate in the study.

A multidisciplinary team from the Regional Centre for Autism of the Abruzzo Region, Italy, with experience in ASD diagnosis, performed the neuropsychiatric assessment based on standardised measures and clinical observations according to the criteria of the *Diagnostic and Statistical Manual of Mental Disorders* (5th ed.; *DSM-5*; 1). Evaluation of ASD symptoms and the child’s functional development was conducted by a trained clinical psychologist (please refer to the ‘Measures’ section). After the evaluation, caregivers were provided with assessment outcomes, including diagnosis and recommendations for intervention.

### Participants

The inclusion criteria were patients aged between 18 and 48 months at their first clinical consultation, the availability of the GMDS at both baseline and follow-up (i.e. T0 and T1) and confirmation of the diagnosis if they were considered at risk in the first evaluation. Children who presented other significant medical conditions (e.g. other significant genetic disorders, other neurological disorders, epilepsy, significant hearing and visual sensory deficits, traumatic brain injury) were excluded. A total of 79 patients were recruited for the study and followed for ASD symptoms between June 2019 and November 2020. Four participants were excluded because the diagnosis was not confirmed at T1, and eight participants dropped out for various reasons. The final research sample was 67 children with a mean age of 33.08 months (*SD* 8.20 months), composed of 45 males (67.2%) and 22 females (32.8%). The GMDS Practical Reasoning scale was not administered to all children at T0 as it cannot be administered at an age below 24 months, as specified in the Measures section. Furthermore, because the scale is complex, some children were not able to take it and were not included in the measurement. Thus, the data from this scale are available for 38 subjects from the total sample at T0. The assessment of the scale is therefore available for the total sample at T1 (after 12 months). Demographic and clinical data of the participants for each timepoint are reported in Table 1.

**Table 1:** Descriptive statistics and clinical data of participants at each time point (N = 67)

	T0 Mean (SD)	T1 Mean (SD)	Mean ΔT (SD) [range]
Mean chronological age in months (SD)	33.08 (8.20)	45.65 (8.52)	12.57 (1.33) [10–14]
Gender (M;F)	45;22	45;22	
Race/Ethnicity			
Caucasian	100%	100%	
ADOS-2 toddler module [calibrated score (SD)]			
SA	14.72 (5.06) [7.78 (2.37)]	–	
RRB	3.17 (2.12) [6.78 (2.21)]	–	
Total	17.89 (6.46) [7.89 (2.59)]	–	
ADOS-2 Module 1 [calibrated score (SD)]			
SA	12.98 (4.76) [5.17 (2.48)]	12.68 (5.62) [6.88 (2.68)]	
RRB	3.26 (2.16) [6.50 (2.75)]	3.29 (2.26) [6.31 (2.77)]	
Total	16.28 (5.93) [5.44 (2.24)]	15.97 (6.83) [6.80 (2.62)]	

*SA* social affect score, *RRB* restricted and repetitive behaviour score, *Total* total score;

### Measures

#### ADOS-2

The ADOS-2 [40] is viewed as ‘the gold standard’ for observational assessment of ASD. This instrument provides a highly accurate picture of current symptoms and does not depend on language. It can be used to evaluate almost anyone suspected of having ASD—from 1 year of age with no speech, to adults who are verbally fluent. The ADOS-2 is a semi-structured, standardised assessment of communication, social interaction, play and restricted and repetitive behaviours. It presents various activities that elicit behaviours directly related to a diagnosis of ASD. By observing and coding these behaviours, one can obtain information that informs the diagnosis, treatment planning and educational placement. It includes five modules, each requiring just 40 to 60 min to administer. The individual being evaluated is given only one module, selected based on his or her expressive language level and chronological age. Following guidance provided in the manual, you choose the module that is appropriate for the individual you are evaluating. In addition to a total score, the ADOS-2 provides scores for Social Affect (SA) and Restricted and Repetitive Behaviour (RRB). According to Esler et al. [41] for Toddler Module and Hus et al. [42] for Module 1, we separately calibrated raw totals from the ADOS SA and RRB domains, used in our analysis.

### Assessment of Mental Development with the GDMS

The GMDS [30, 31] was developed as a child development assessment tool for children aged 0–24 months. The extended form can be used up to the age of 8 years. Hence, the instrument consists of two scales: the 0–2 scale and the 2–8 scale. The GMDS 0–2 and the extended form have been revised, leading to the development of the version for children up to 8 years of age: GMDS-ER [43, 44]. These scales measure a child’s abilities through reference to the following six sub-scales: sub-scale A, Locomotor, measuring postural development, deambulation and higher-order motor skills; sub-scale B, Personal-Social, concerning the subject’s ability to adapt, personal autonomy and social interaction skills; sub-scale C, Hearing and Language, tracing the main stages of linguistic and communicative development and assessing attention to sounds, production of vocalisations and early lexical development; sub-scale D, Eye and Hand Coordination, assessing visual control and fine-motor dexterity skills; sub-scale E, Performance, concerning visual perception awareness, including working speed and accuracy; and sub-scale F, Practical Reasoning, from the age of 2 years, it consists of items that assess the ability to use knowledge learned from the environment to solve problems and to understand mathematical concepts and moral problems.

The GMDS raw score obtained from the sum of the items passed in each scale can be used to calculate the sub-quotients and a general quotient (GQ). The sub-quotients and GQ show a deviation from the mean for each month of age, for each sub-scale and the total score. The scores are calculated by using the developmental age corresponding to each sub-scale divided by the actual chronological age and multiplying by 100. The mean of the six sub-quotients and the GQ is 100 points (*SD* 16).

### Statistical Analysis

A repeated measures *t*-test was performed to determine whether there was a difference between T0 and T1 samples of the GMDS sub-scales and GQ to understand whether any of the abilities had worsened during the time interval considered. Cohen’s *d* was calculated to assess the effect size. The alpha level was set at 5% multiple comparisons were adjusted by Bonferroni method. Accordingly, the p-values associated with the statistical tests carried out have been inflated by the factor 7, representing the number of comparisons.

To explore the association between GDMS and gravity of the symptoms, we performed multivariate regression analysis. Specifically, multivariate regression allows one to assess whether more than one independent variable (X_i_) could predict a dependent variable (Y). The advantage of using multivariate regression is that it could account synchronically for the variation in the independent variables in the dependent variable, thus allowing a better estimation than considering independent variables individually. Specifically, we assessed whether the calibrated SA and RRB scores of the ADOS-2, at T1 could be affected by the GMDS measured at the same time. Multicollinearity was detected by using the variance inflation factor (VIF), where values < 10 were considered acceptable [45].

## Results

### Differences in the GDMS Abilities Between Time Points

**Fig. 1:**
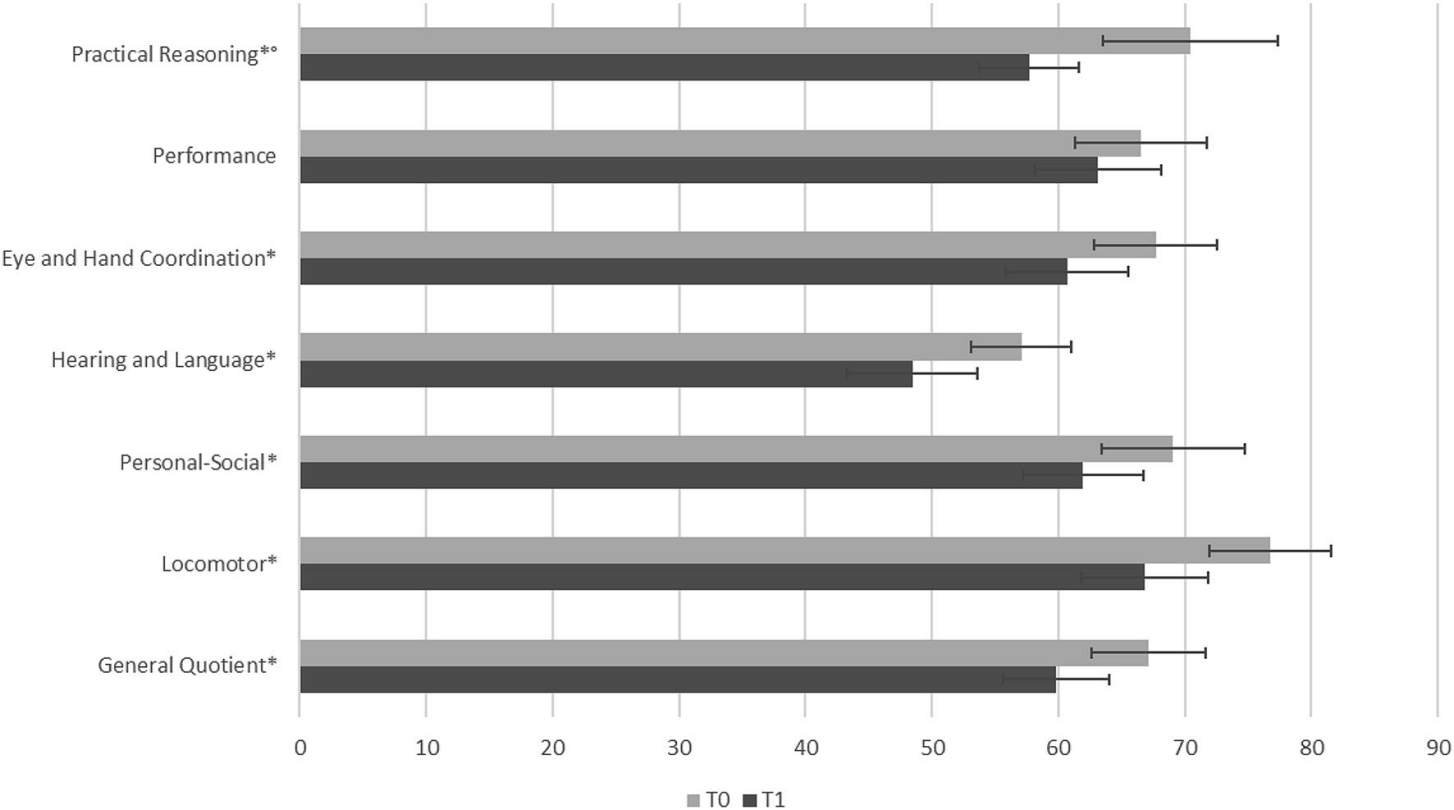
Means (± 2 SE) of GDMS scores of T0 and T1. *p < .05. Practical Reasoning comparison was performed on 38 participants at T0. It should be noted that the Practical Reasoning scale cannot be administered to an age below 24 months

A repeated measures *t*-test revealed significant reductions in GMDS abilities between T0 and T1 (Table 1). Specifically, Locomotor abilities decreased significantly from T0 (*M*_0_ = 76.78, *SD*_0_ 20.57) to T1 (*M*_1_ = 66.85, *SD*_1_ 19.87; *t*(66) = 4.84, *p* < 0.001, *d* = 0.59); Personal and Social abilities decreased significantly (*t*(66) = 3.23, *p* = 0.014, *d* = 0.39) between T0 (*M*_0_ = 69.07, *SD*_0_ 19.53) and T1 (*M*_1_ = 61.93, *SD*_1_ 23.27); Hearing and Language abilities decreased significantly (*t*(66) = 3.99, *p* < 0.001, *d* = 0.49) between T0 (*M*_0_ = 57.07, *SD*_0_ 21.26) and T1(*M*_1_ = 48.45, *SD*_1_ 16.12); Eye and Hand Coordination decreased significantly (*t*(66) = 3.26, *p* = 0.014, *d* = 0.40) between T0 (*M*_0_ = 67.69, *SD*_0_ 19.92) and T1 (*M*_1_ = 60.67, *SD*_1_ 19.94); and Practical Reasoning decreased significantly (*t*(37) = 3.85, *p* < 0.001, *d* = 0.63) between T0 (*M*_0_ = 70.47, *SD*_0_ 12.35) and T1 (*M*_1_ = 57.66, *SD*_1_ 21.34). Performance abilities did not change between T0 and T1. The GQ measured from the GMDS showed a significant decrease from T0 (*M*_0_ = 67.13, *SD*_0_ 16.82) to T1 (*M*_1_ = 59.78; *SD*_1_ 17.97; *t*(66) = 3.98, *p* < 0.001, *d* = 0.50). Results are shown in Fig. 1.

### Multivariate Linear Regression

The regression model showed that the scores of the GMDS scales at T1 significantly predict the calibrated SA score of the ADOS-2 measured at the same evaluation, showing a good fit of the data and explaining 47% of the variance (*F*(5, 62) = 8.93, *p* < 0.001, *R*^2^ = 0.47, f^2^ = 0.88). Among the independent variables, we found Personal and Social skills (β =  − 0.04, *SE* = 0.02, *t* =  − 2.21, *p* = 0.031) and Practical Reasoning skills (β =  − 0.07, *SE* = 0.02, *t* =  − 3.80, *p* < 0.001) as significant predictors. Thus, keeping the other variables constant, these results indicate that for each sub-quotient point increase in Personal and Social skills we could expect a decrease of 0.04 in the calibrated SA score, and for each sub-quotient point increase in practical reasoning skills we could expect a decrease of 0.07 in the calibrated SA score.

**Fig. 2:**
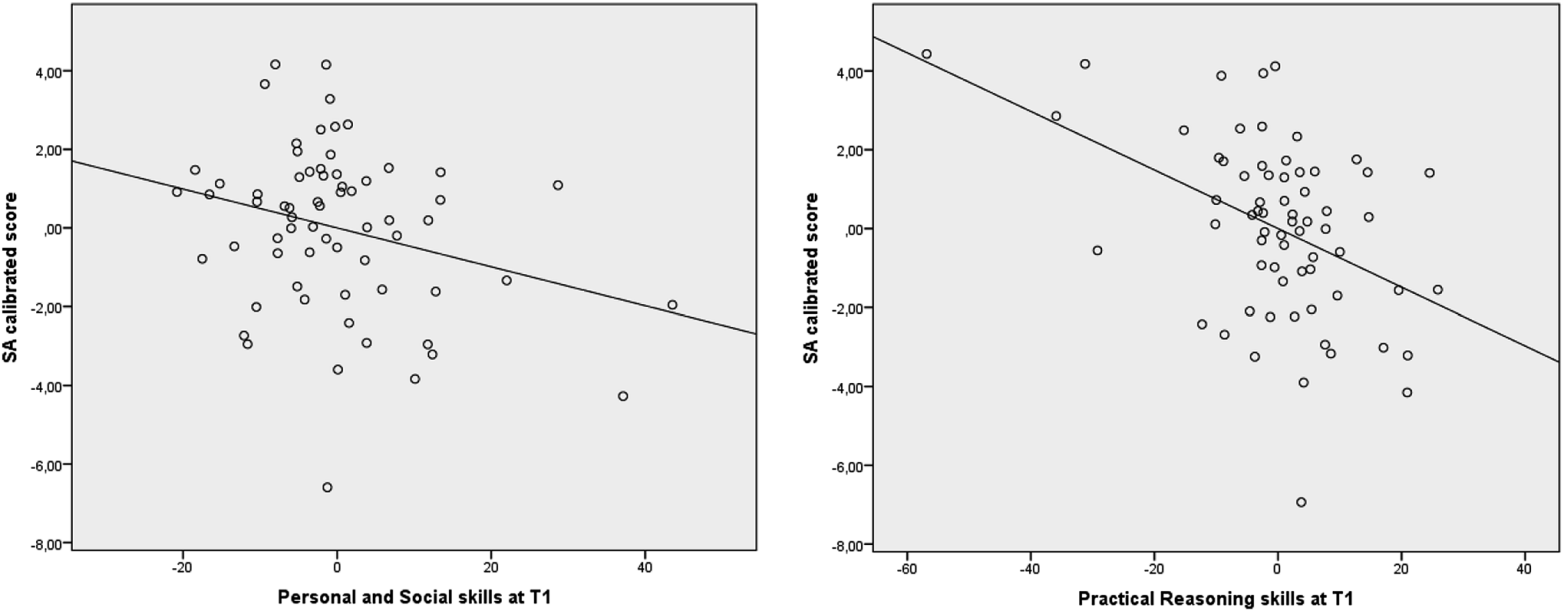
Partial regression plot of GDMS skills at T1 significantly predicting SA score

**Fig. 3:**
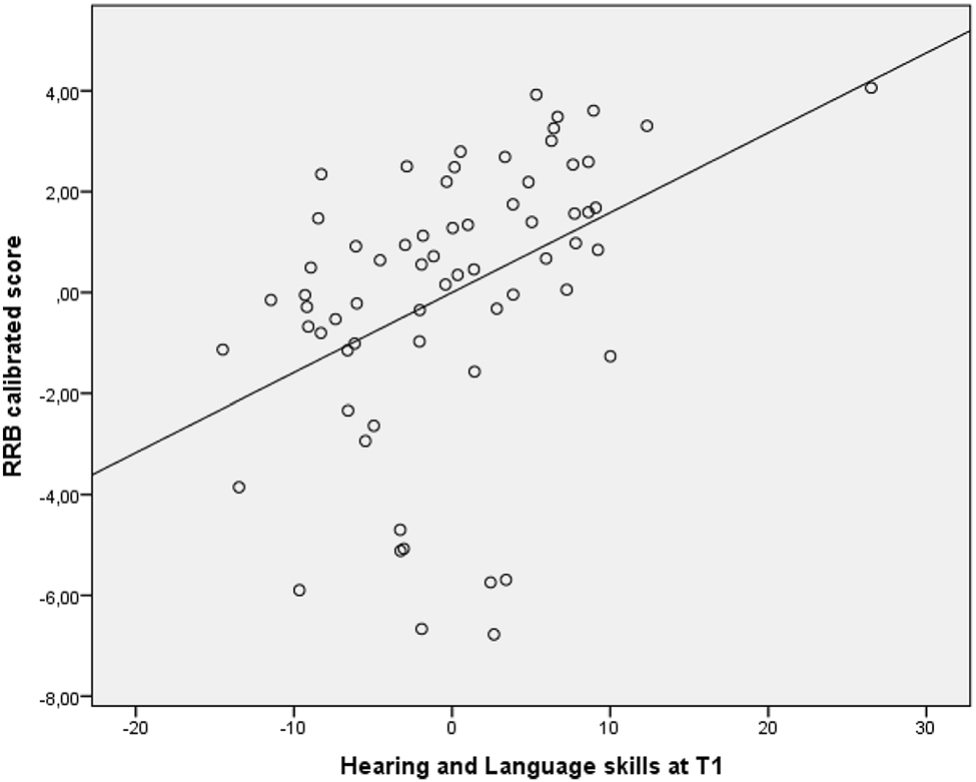
Partial regression plot of GDMS skills at T1 significantly predicting RRB score

The regression model also significantly predicts the calibrated RRB score of the ADOS-2 measured at the same evaluation, explaining 27% of the variance (*F*(5,62) = 3.54, *p* = *0.005*, *R*^2^ = 0.27, f^2^ = 0.37), where Hearing and Language skills (β = 0.15, *SE* = 0.004, *t* = 3.61, *p* = 0.001) represent significant predictors of the RRB score. These results indicate that for each unit increase in hearing and language skills we could expect an increase of 0.15 in the RRB score. The results are shown in Figs. 2 and 3.

## Discussion

We assessed the rate of development, through the GMDS, in children with ASD. Specifically, we evaluated which abilities from the GMDS could be associated to symptomatologic severity typical of ASD. We know that the assessment of child development (through screening for developmental indicators) allows the recognition of delayed or at-risk children; thus, collecting information during clinical evaluation is an important step to support an early diagnosis and planning of an intervention programme. Increased interest in early diagnosis and intervention has also led to increased knowledge about the prediction of ASD symptoms and their level of severity through the assessment of other skills (motor, language or social abilities).

We implemented a longitudinal observational study in which we first assessed the GMDS in children at risk or with a diagnosis of ASD (T0); we then performed a follow-up of the GMDS assessment after 12 months and collected the ADOS-2 scores for all participants, confirming the diagnosis where needed (T1). The aim of our analysis was to determine whether there was a difference between T0 and T1 sample observations in the GMDS sub-scales to determine if any of the abilities measured by the instrument was changed after 1 year. In addition, we investigated whether the GMDS scores could predict the severity of symptoms. From T0 to T1, we found a developmental delay of the GQ and all sub-quotients measured by the GMDS, except for the Performance sub-quotient. In general, this translates into the fact that the children in our sample acquire, over time, fewer skills investigated by the GMDS than expected levels (normative test values). Our results did not find significant differences regarding the Performance sub-quotient which evaluates visuospatial skills including execution speed and accuracy [44]. This result is in line with a previous study that indicated that Performance is a relative point of strength for ASD [39]. This subscale requires detecting, processing, and memorizing visual stimuli. ASD could demonstrate better performance, compared to typical peers, in the processing of visual material [46], and this argument has been long discussed [47, 48]. Better performances in visual paradigms have been attributed to a detail-oriented processing style or an “enhanced perceptual function” on low-level visual tasks [49]. Typical autistic characteristics such as systemizing and attention-to-detail have been associated with better visuospatial abilities during figure dissembling and mental rotation tasks [50, 51]. Thus, these findings could be due to the ASD-specific style of processing visual material. Our results extend previous knowledge, as it seems that competencies in performance skills tend to be maintained during the development, representing their point of strength during childhood.

Our second aim was to analyse which skills from the GMDS could be associated the severity of symptoms that characterise ASD. We found that three sub-quotients—Personal-Social, Hearing and Language and Practical Reasoning—are associated with symptom severity at the time when the diagnosis of ASD is made. The significant results obtained from regression of the three sub-quotients are discussed below.

### Personal-Social

The Personal-Social scale assesses the child’s abilities in activities of daily living and the ability to interact with other children. We found that the Personal-Social skills measured by the GMDS affect ADOS-2 outcomes. Specifically, an increase of 1 point in this sub-scale could lead to a decrease in the calibrated SA score. The result obtained for this scale is not surprising, as a deficit in social skills is the core symptom in ASD [13, 18]. In particular, the Personal-Social scale includes items (e.g. ‘smiles’, ‘follows moving people with his eyes’, ‘claps in imitation’) that assess social learning and the acquisition of social habits, indicative of this stage of personal-social adaptation. Social experiences contribute to the development of a set of cognitive processes that enable individuals to interact appropriately with their environment [52].

From birth, infants appear to be predisposed to develop various social skills, such as the ability to identify social agents, mutual affiliation and a preference for social over non-social models [45]. The set of cognitive processes that drive social skills are grouped under the name social cognition. Many researchers [13, 53, 54] have shown that these skills, including theory of mind, are evolutive skills that develop along a continuum, following specific stages. In ASD, there is an alteration in the timing of the acquisition of functions and precursors underlying the formation of social cognitive skills [13, 55]. Although detecting social deficits in the first year of life can be challenging, there is growing evidence that children subsequently diagnosed with ASD show a reduction in attention to social scenes as early as 6 months [56]. Other indicators have also been reported as early signs of social problems that are present in early childhood, such as social interest, shared affection, looking at faces and eyes and responding to one’s name [14].

### Hearing and Language

With this scale the examiner is able to assess the child’s receptive and expressive language skills. It includes items such as naming colours and objects, repeating sentences and answering questions that test the child’s comprehension. The relation between this sub-scale measured at T1 and the calibrated RRB score of the ADOS-2 is of particular interest. The regression analysis showed that acquisition of language skills also leads to an increase in the RRB score, thus influencing symptom severity. Although this finding could be considered a counterintuitive outcome, this result can be traced back to the characteristics of the ADOS-2 diagnostic tool. In fact, during the ADOS-2 assessment, the examiner selects the most appropriate module on the basis of the level of expressive language. In particular, children in our sample were assessed using Module 1, which is intended for children aged 31 months and over who possess verbal abilities ranging from no verbal language to simple sentences [40]. Within the assessment, the presence of restricted and repetitive behaviour in children with ASD is also evidenced by the stereotyped use of words or phrases, as well as by the intonations of vocalisations and verbalisations. Coding for this includes the use of deferred echolalia or highly repetitive expressions, such as words or phrases that are understandable and may be appropriate to the conversation, focusing on the stereotypical quality of the sentence or the unusual use of words. This makes it clear that the assessment of language use is possible when the child begins to express themselves verbally. Another possible consideration concerns the coding and scoring of this characteristic. In the instrument, there is a coding of 8 (i.e. not applicable), which is converted to 0 when the examiner has to count the final score. This coding, in reality, represents a lack of information and influences the total RRB score; thus, for children with very low language skills for whom the items regarding repetitive language are not applicable, their RRB score is likely to be reduced by the inclusion of a score of 0. There are also studies in the literature showing that the expressive language skills of children with ASD increase significantly from infancy to primary school; however, difficulties remain in pragmatic and semantic features [57]. These difficulties are reflected in children’s attempts to initiate social interactions; to tell stories, events or experiences; and by the occurrence of certain unusual features (e.g. immediate echolalia, stereotyped language). The non-spontaneous and functional use of language adds important information for the development of specific intervention programs for children with ASD.

### Practical Reasoning

The Practical Reasoning scale is administered from 24 months onwards. It investigates the child’s ability to solve practical problems and incorporates mathematical concepts as well as ethical and moral issues that require greater linguistic comprehensibility. Our results show a relationship between the acquisition of these skills and a lower score for communication and social interaction difficulties investigated by the calibrated SA score of the ADOS-2. This scale requires the acquisition of those skills and general cognitive functioning, such as reasoning, attention, learning and memory. These skills represent a set of higher-order processes that influence social competence [59] and can be encapsulated under the umbrella term of executive functions. Executive functions can have an impact on social competence by negatively affecting processes that involve theory of mind skills and social recognition [58–62]. Thus, as our results show, working on the acquisition of those cognitive skills that allow the child to solve practical problems consequently influences the skills needed for social engagement. Interventions on social skills in children with ASD should be aimed at identifying and enhancing key components of executive functioning that influence social competence in children with ASD.

Previous studies [6, 26, 58, 63] have shown an association between cognitive skills and autistic symptoms; however, in this study, our results show that it is the development of these skills that is associated with ASD symptoms. Knowing how and when a child acquires these skills means being able to intervene early to modify atypical developmental trajectories and improve prognosis. Compared to previous studies, our research focus takes a dynamic approach—that is, we expect changes in acquired abilities depending on the severity of the symptomatology or the use of specific, individualised interventions. Development is a dynamic process, so the study of the development of skills in children cannot be limited to a static view (i.e. to a single assessment of a moment), but to monitoring over time.

### Limitations and Future Perspective

We think that the most important limitation in our study concerns T0, because in this first assessment we not only included subjects at risk, but also children with a certain diagnosis. For this reason, in the future we think it is necessary to consider children under 30 months of age who do not yet have a diagnosis and to determine if indeed the GMDS evaluation could affect the diagnosis of ASD. Moreover, at present the updated version of the GMDS is version III, but at the time of our data collection we did not have this version of the test available.

Another limitation regards RRB scores of ADOS-2. Although in our study we use Module Toddler and Module 1, Kuhfeld and Sturm [64] have demonstrated that RRB measured with the 3 and 4 Modules are an unreliable measure of this construct. Thus, we suggest having some caution in interpreting results related to RRB. We also used the calibrated severity score of two domains of ADOS-2 (AS and RRB); the calibrated severity score is not a continuous indicators of autism symptom severity, and this represents another limitation of this study.

In addition, a procedural limitation regards the limited developmental time period over which children were assessed (1 year).

It is unclear from our study, if the observed uniformity of development delay in GMSD domains in ASD, indicates a true decrease in ability, or it is artifact of the measure itself. Consequently, further investigations are requested. Our future perspective is to overcome these limits to identify a typical developmental profile for ASD. In addition, we would like to evaluate how cognitive abilities, in a larger sample ASD, change over time to make possible differential diagnosis with other neurodevelopmental disorders.

## Summary

In this study we focused on two aims: (a) to assess longitudinally whether children diagnosed with ASD show a worsening of abilities measured by the GMDS over time and (b) to analyse which skills of the GMDS could be associated with the symptomatologic severity typical of ASD.

Our results extend those obtained from a previous study [39], in which the authors reported a relationship between the symptomatic severity of ASD and different levels of development in various areas of cognitive structures. In fact, our aim was to evaluate longitudinally the cognitive development of children who had been diagnosed with ASD, highlighting how developmental delay in certain abilities investigated by the GMDS could be associated with the symptomatologic score of the ADOS-2, which is considered the gold standard for diagnosis. Our study shows that children with ASD present an impaired acquisition of most of the developmental skills measured with the GDMS. Moreover, we found that Personal-Social, Practical Reasoning and Hearing and Language skills are associated with autism severity, predicting the ADOS-2 scores.

We believe that our results are of relevance in clinical and rehabilitation practice. Indeed, knowing how and when children acquire the prerequisites for the development of more complex skills also allows clinicians to plan targeted and individualised interventions, thus improving prognosis. A late diagnosis of ASD has a negative impact on the developmental outcome and learning of these children. Therefore, it is crucial to emphasise the importance of providing better diagnosis and intervention structures and learning strategies for children with ASD.
